# Repeat low-level blast exposure increases transient receptor potential vanilloid 1 (TRPV1) and endothelin-1 (ET-1) expression in the trigeminal ganglion

**DOI:** 10.1371/journal.pone.0182102

**Published:** 2017-08-10

**Authors:** Elaine D. Por, Melody L. Sandoval, Chiquita Thomas-Benson, Teresa A. Burke, Allison Doyle Brackley, Nathaniel A. Jeske, Jeffery M. Cleland, Brian J. Lund

**Affiliations:** 1 Ocular Trauma, United States Army Institute of Surgical Research, Fort Sam, Houston, Texas, United States of America; 2 Department of Pharmacology, University of Texas Health Science Center at San Antonio, San Antonio, Texas, United States of America; 3 Department of Oral and Maxillofacial Surgery, University of Texas Health Science Center at San Antonio, San Antonio, Texas, United States of America; 4 Department of Physiology, University of Texas Health Science Center at San Antonio, San Antonio, Texas, United States of America; Indiana University School of Medicine, UNITED STATES

## Abstract

Blast-associated sensory and cognitive trauma sustained by military service members is an area of extensively studied research. Recent studies in our laboratory have revealed that low-level blast exposure increased expression of transient receptor potential vanilloid 1 (TRPV1) and endothelin-1 (ET-1), proteins well characterized for their role in mediating pain transmission, in the cornea. Determining the functional consequences of these alterations in protein expression is critical to understanding blast-related sensory trauma. Thus, the purpose of this study was to examine TRPV1 and ET-1 expression in ocular associated sensory tissues following primary and tertiary blast. A rodent model of blast injury was used in which anesthetized animals, unrestrained or restrained, received a single or repeat blast (73.8 ± 5.5 kPa) from a compressed air shock tube once or daily for five consecutive days, respectively. Behavioral and functional analyses were conducted to assess blast effects on nocifensive behavior and TRPV1 activity. Immunohistochemistry and Western Blot were also performed with trigeminal ganglia (TG) to determine TRPV1, ET-1 and glial fibrillary associated protein (GFAP) expression following blast. Increased TRPV1, ET-1 and GFAP were detected in the TG of animals exposed to repeat blast. Increased nocifensive responses were also observed in animals exposed to repeat, tertiary blast as compared to single blast and control. Moreover, decreased TRPV1 desensitization was observed in TG neurons exposed to repeat blast. Repeat, tertiary blast resulted in increased TRPV1, ET-1 and GFAP expression in the TG, enhanced nociception and decreased TRPV1 desensitization.

## Introduction

The increased incidence of blast-related sensory and cognitive injuries sustained by military personnel has led to extensive research focused on characterizing the pathophysiology underlying these distinct injuries. Importantly, current research efforts have shifted from severe and penetrating injuries to closed brain and mild traumatic brain injuries (TBI), which include high rates of sensory impairment, pain and polytrauma that are not usually associated with typical traumatic injuries such as motor vehicle accidents or falls [1]. Military service members injured by blast have a broader spectrum of physical injuries, higher levels of admission and discharge opioid analgesic use, reduced improvement in pain intensity following treatment, and much higher rates of posttraumatic stress disorder (PTSD) and other psychiatric diagnoses [2–4]. Although the effects of blast exposure on the central nervous system is an active area of investigation, additional research is required to appropriately characterize the molecular mechanisms that underlie blast injuries sustained by military personnel and specialized law enforcement units. The incidence of blast exposure and category of blast are critical factors to consider as both variably impact trauma severity and differentially disrupt normal physiology and molecular signaling pathways. With these critical factors taken into consideration, identification of proteins activated and/or up-regulated following blast exposure may potentially lead to therapeutic targets for traumatic and debilitating blast injuries, to include chronic pain, migraines and photophobia.

Blast injuries are generally categorized as primary to quaternary and evoke a myriad of effects on multiple body systems, to include pulmonary contusions, bone fractures and neurological damage [5–7]. Primary blast injuries are due to direct effects of the blast wave, secondary injuries result from fragments or other debris thrown by the blast that strike an individual, tertiary injuries involves displacement of the entire body and impact with other objects, and quaternary refers to other effects such as heat, chemical, or electromagnetic wave generation [5–7]. Dependent on the blast exposure and injuries sustained the severity, subsequent recovery and rehabilitation can be vastly different. Recent findings from our group have characterized the effects of single and repeat low-level blast on ocular tissues [8] and have shown increased apoptosis in the optic nerve and retinal tissues following blast exposure [9]. Importantly, additional findings have also shown that low-level blast exposure resulted in increased expression of pain signaling proteins and inflammatory cells in the cornea [10]. Specifically, increased expression of transient receptor potential vanilloid 1 (TRPV1) and endothelin-1 (ET-1) was revealed in the epithelial and stromal layers of the cornea exposed to repeat blast [10]. TRPV1 expression is well characterized for its role in mediating pain transmission, while ET-1 is known to potentiate TRPV1 activity and receptor-mediated nocifensive behavior [11–13]. Based on these findings, we sought to further investigate the effects of low-level blast exposure on pain signaling processes. Specifically, we aimed to investigate the effects of single and repeat blast on ocular associated sensory tissues as well as differentiate the functional consequences of primary and tertiary blast.

The TRPV1 receptor is expressed in various tissues of the eye including corneal epithelial, endothelial layers, stromal fibroblasts and nerve fibers [14, 15]. Recent literature also indicates a role for TRPV1 in the eye in wound healing and neuronal injury (25). Importantly, TRPV1 is well characterized for its involvement in pain signaling and its expression in the trigeminal nerve, the principal sensory nerve of the head that provides dense sensory input to the cornea. Recent literature has established a correlation of increased TRPV1, calcitonin gene-related peptide (CGRP) and substance P (SP) expression levels with patients suffering from chronic migraines [16, 17]. A significant percentage of military veterans with blast-related injuries and confirmed TBI have been diagnosed with migraine headaches and/or chronic, daily headaches [18]. Moreover, a recent retrospective case study also confirmed that a common complaint of patients with mild TBI is photophobia [19], a hallmark symptom of migraines and other neuro-ophthalmic disorders. Post-traumatic photophobia is intimately linked to pain sensation and involve the activation of intraocular afferents and trigeminal neurons [20, 21]. Unfortunately, the precise biochemical and molecular alterations that underlie blast-related injuries is not well characterized. Thus, we sought to expand our research efforts and investigate primary and tertiary blast effects on pain and inflammatory processes with our rodent blast model. A greater understanding of blast-related trauma is required to effectively manage and treat secondary blast injuries that affect the respective sensory systems.

Our findings in this study revealed that unrestrained animals exposed to repeat, tertiary low-level blast demonstrate increased TRPV1 expression in the trigeminal ganglion (TG) and decreased receptor desensitization. Increased ET-1 expression was also detected in the TG and blood plasma following repeat, tertiary blast. The data presented herein indicate exposure to a repeat, tertiary blast increased co-expression of TRPV1 and the ET-1 receptor subtype, ET-A, in the TG. Enhanced TRPV1 activity was supported by increased CGRP and SP expression in the TG following repeat, tertiary blast. Moreover, increased glial fibrillary associated protein (GFAP) expression detected in the TG suggests that blast exposure resulted in glial activation. Altogether, these evidence demonstrate that the cumulative effects of repeat, tertiary blast exposure leads to up-regulation of key proteins involved in pain and inflammation signaling. Additional lines of investigation are necessary to determine the functional impact of these protein alterations with regard to clinical diagnoses of blast-related injuries such as chronic pain, migraine and photophobia.

## Methods

### Blast exposure experimental design

**Fig 1:**
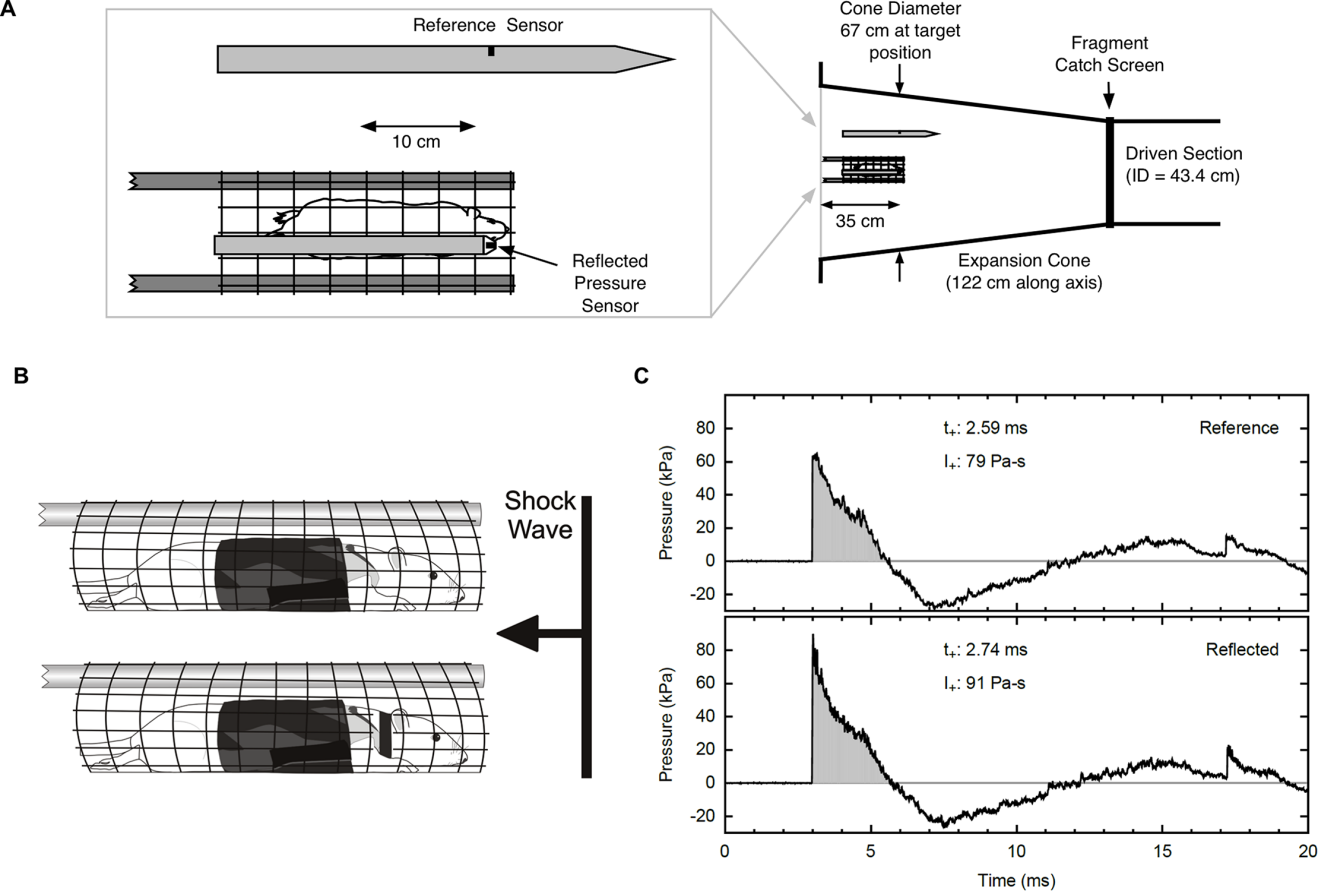
Blast exposure experimental design. (A) Top view of animal in expansion cone of shock tube with positions of reference and reflected sensors indicated. Diagram illustrates length of driven section and expansion cone of shock tube and cone diameter at target position. (B) Side view of animal with Kevlar vest in mesh holder. Top diagram illustrates unrestrained animal group and bottom diagram illustrates restrained animal group. (C) Representative shock wave produced by the USAISR shock tube as measured by the reflected pressure and overpressure sensors. Pressure represented in kilopascal (kPa). (t_+_ = time; duration of peak, I_+_ = impulse; area under peak).

Adult male Long Evans rats, 300–350 g, (Envigo; Houston, TX) were randomly divided into one of four blast-exposure groups (single unrestrained, single restrained, repeat unrestrained, and repeat restrained) or a control group for the single blast and repeat blast (n = 5 per group). Animals were anesthetized with a ketamine/dexmedetomidine (50/0.25 mg/kg) cocktail, and positioned into a secured holder that prevented animal exposure to fragments and/or debris as previously described [10]. Anesthetized rats were placed in the prone position, head facing the blast front, on a holder consisting of a flat plastic mesh (Tenax Cintoflex E mesh; Tenax Corporation, Baltimore, MD) suspended between two stainless steel rods, which run horizontally alongside the animal. A second plastic mesh was placed over the top of the rats and secured to rods to prevent the animals from falling off the holder (Fig 1). Mesh openings were approximately 0.5” x 0.6”. Thoracic protection was provided by a vest fashioned from Kevlar as previously described in Long, et al [22].

Rats were exposed to shock waves using a compressed air-driven shock tube (Applied Research Associates; Littleton, CO). The driver and driven sections of the tube were constructed from 17 inch inner diameter steel pipe. The target area was within an expansion cone mounted at the end of the driven section of the tube (Fig 1B). A fragment catch screen mounted between the driven section and the expansion cone prevented large pieces burst disks from reaching the target. Burst disks were cut from sheets of 0.016 inch thick 6061 T6 aluminum (Aircraft Spruce & Specialty Co.; Corona, CA). A model XT-190-100SG pressure transducer (Kulite Semiconductor Products, Leonia, NJ) was mounted with its sensitive element facing into the oncoming shock wave to measure reflected pressure near the target position (Fig 1B). A model 137A23 Pencil Probe (PCB Piezotronics; Depew, NY) was used as a reference sensor to monitor the overpressure of the blast wave. The reference sensor was mounted some distance away from the target, to minimize reflections back toward the test subject. Prior to placing animals in the shock tube, the reference sensor was calibrated against a Kulite model XTEL-190-50A pressure transducer, mounted in a 6-inch diameter beveled disk and placed in the target position within the expansion cone.

Rats in this study were subjected to a peak overpressure of 73.8 ± 5.5 kPa for a positive pressure peak duration of 2.7 ± 0.1 msec for positive phase duration as previously described [23]. Fig 1C is a representative shock wave produced by the USAISR shock tube as measured by reflected pressure and overpressure (reference) sensors. Prior to placing animals in the shock tube, the overpressure sensor was calibrated against another sensor placed at the target position. Control animals were handled in the same manner without blast exposure, in which they were anesthetized and placed in the shock tube without firing. Blast exposed rats received either a single blast exposure on the first day of experiments or repeat blast exposure once daily for five consecutive days. All single and repeat blast-exposed groups were euthanized on day 5 or 9 of the experiment, respectively (i.e. four days after the final blast wave exposure).

To evaluate primary blast effects, the rat head was secured to the mesh (restrained) with a phlebotomy tourniquet positioned behind the ears and fastened under the jaw (Fig 1A, bottom diagram). The tourniquet was applied with care to ensure it was not constrictive but functioned to minimize movement of the head in response to blast exposure. To evaluate tertiary blast effects, rats were not secured to the mesh (unrestrained) and the head was allowed to recoil in response to the blast wave (Fig 1A, top diagram). Intraocular pressure (IOP) measurements were taken immediately before and after blast exposure with a rodent tonometer (iCare Tonolab; Raleigh, NC) to monitor pressure of all animal groups. This study was conducted in compliance with the Animal Welfare Act, implementing Animal Welfare Regulations, the principles of the Guide for the Care and Use of Laboratory Animals and approved by the U.S. Army Institute of Surgical Research (ISR) Institutional Animal Care and Use Committee (IACUC).

### Western blot analysis

For Western blotting, frozen trigeminal ganglion (TG) were homogenized via Bullet Blender (Next Advance Inc.; Averill Park, NY) and lysed in RIPA buffer with protease inhibitors (Santa Cruz; Santa Cruz, CA). Protein quantification was performed using the bicinchoninic acid (BCA) protein assay (Thermo Scientific; Waltham, MA). (Quantified values for protein analysis provided in the S1 Supporting Document). Protein samples (50 μg) were resolved via 4–12% gradient acrylamide SDS-PAGE and transferred to nitrocellulose membranes (NuPage Novex, Life Technologies; Grand Island, NY). Western blots were blocked in 5% nonfat milk in Tris-buffered saline/Tween 20 and visualized using a TRPV1 primary antibody (R130; Santa Cruz, CA) followed by the appropriate horseradish peroxidase-conjugated secondary antisera and enhanced chemiluminescence (ECL) detection following the manufacturer’s instructions (Biorad, Hercules, CA).

### Immunohistochemistry

Hematoxylin and Eosin (H&E) staining was conducted on frozen TG tissue sections (5μm) to visualize ganglion structure. Briefly, slides were incubated in Harris’ Hematoxylin for 5 min, rinsed in H_2_0 for 5 min and differentiated in acid alcohol (1% HCl in 70% ethanol) for 10 sec. Next, sections were rinsed with distilled H_2_0 (dH_2_0) for 5 min, incubated in ammonia water (0.25% ammonia hydroxide in dH_2_0) for 3 min and rinsed with dH_2_0 2X’s (5 min). Sections were dipped in alcoholic Eosin for 10 sec, rinsed with dH_2_0 2X’s (5min), dehydrated in ethanol and then mounted onto coverslips (Permount, Fisher Scientifc; Hampton, NH).

Protein expression was also determined in frozen TG sections using an antibody directed against glial fibrillary associated protein (GFAP) (Z033429-2, Agilent Technologies, Santa Clara, CA). A standard immunohistochemical avidin-biotin-peroxidase complex technique (Vectastain Elite ABC kit, PK-6100, Vector Laboratories, Burlingame, CA) was used to visualize protein expression using 0.02% of 3-diaminobenzidine tetrahydrochloride. Sections were then counterstained with methyl green. Images were acquired with an Olympus BX3 microscope equipped with a DP73 17.28 megapixel digital color camera using a 20X objective (Olympus Life Science; Center Valley, PA).

### Immunofluorescence

Protein expression was determined in frozen TG tissue sections using antibodies directed against TRPV1, CGRP, (GP14100; RA24112, Neuromics, Edina, MN), ET-1, ET-A and SP (ab2786, ab117521; ab14184, Abcam, Cambridge, MA). Following primary antibody incubation, tissue sections were incubated with the appropriate Alexa-Fluor 568 and 488 secondary antibodies (Life Technologies; Grand Island, NY). Sections receiving only secondary antibody were included as negative controls. Immunofluorescence images were acquired with an Olympus BX3 microscope equipped with a DP73 17.28 megapixel digital color camera using a 20X objective (Olympus Life Science; Center Valley, PA).

### Capsaicin eye wipe test

To evaluate the effect of blast on ocular pain responses, a dilute capsaicin (CAP) solution 0.02% (saline supplemented with 0.2% ascorbic acid) was applied directly to the eye, as previously described [24, 25]. Prior to anesthesia and blast exposure, behavioral assessments were conducted at time 0, 48, and 96 hrs for single blast animals and 0, 48, 96, 144, and 192 hrs for repeat blast animals. Briefly, the animals’ eyelids were retracted and 50 μL of 0.02% CAP solution was applied directly onto the cornea via pipette. The animal was quickly placed onto a tabletop for observation and eye wipes were counted over the course of 2 min. Nocifensive behavior was recorded as the amount of the time animal spent flinching and/or wiping the affected eye with either the paw or hind leg. To minimize irritation to eye, CAP application was alternated between both eyes of the animal for the indicated testing time points.

### Calcium imaging

Fura-2 AM was used to image individual neurons within a population of cultured TG neurons. Following 2 hr serum-starvation, cells were loaded with fura-2 AM (1 μM; Molecular Probes) in the presence of pluronic F-127 (0.04%; Molecular Probes) for 1 hr at 37°C in the dark, in standard extracellular solution (SES) containing (in mM): 140 NaCl, 54 KCl, 2 CaCl_2_, 1 MgCl_2_, 10 HEPES, 10 D-(+)-glucose, pH 7.40. Cells were viewed on an inverted Nikon Eclipse Ti-U microscope fitted with a 40×/1.35 numerical aperture Fluor objective and imaged using MetaFluor System for Ratio Fluorescence (MetaMorph). Fluorescent images were taken alternately every 3 sec at 340 and 380 nm excitation wavelengths in combination with a 510 nm emission filter with 200-ms exposure. The ratio of ΔF340/F380 was plotted for each cell across time. Intracellular Ca^2+^ levels were analyzed as ΔF340/F380 ratios background corrected and normalized to initial value, R_0_. For each neuron imaged, Ca^2+^ levels were measured for 60 sec under constant SES bath perfusion (<0.01 psi). A multi-barrel glass pipette was used to apply 30 sec exposures of 50 nM CAP at 60 sec and 270 sec. At the end of the experiment, cells were exposed to a depolarizing concentration of KCl (50 mM) for positive neuron identification. (Raw data for individual neuron responses to CAP and KCl are provided in the S1 Supporting Document).

### Enzyme-linked Immunosorbent assay (ELISA)

Concentrations of ET-1 (R&D Systems, Minneapolis, MN) were assessed via ELISA kit in accordance with the manufacturer’s instructions. For ET-1, trunk blood was collected at the time of euthanasia, 96 hrs and 192 hrs, for single and repeat blast animals, respectively. For plasma collection, the blood of each animal was collected in ethylenediaminetetraacetic acid (EDTA)-treated tubes. Cells were removed from blood by centrifugation at 1100 x g for 20 min at 4°C. Analysis of ET-1 expression in blood plasma was assessed from all animal groups. Expression of glial fibrillary associated protein (GFAP) was assessed from homogenized TG lysates (50μg) via ELISA analysis (Millipore; Temecula, CA). TG lysates were prepared as descibed for Western Blot analysis. GFAP expression was assessed from control and repeat, tertiary blast animal groups.

### Statistical analysis

Quantified TRPV1 expression in the rat trigeminal ganglion was performed via one-way ANOVA with Tukey post-hoc analysis. Calcium imaging results and TRPV1 desensitization was analyzed via two-way ANOVA with Bonferroni post-hoc analysis. All data are expressed as the mean ± SEM.

## Results

### Repeat, tertiary low-level blast exposure increase TRPV1 and ET-1 expression in the trigeminal ganglion.

**Fig 2:**
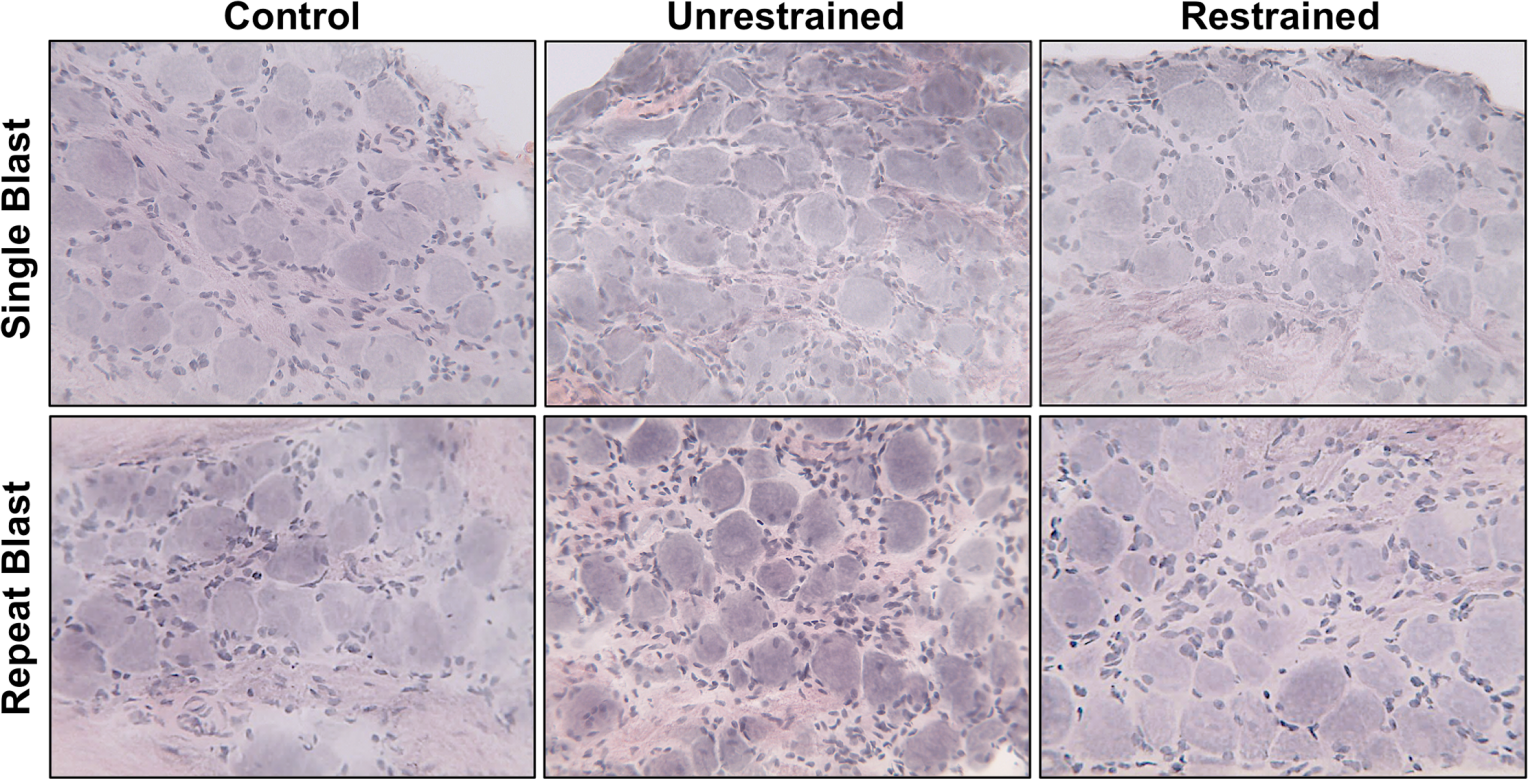
H&E staining of trigeminal ganglion (TG) sections. Frozen sections of TGs, from all animal treatment groups, were subjected to H&E staining to assess ganglion structure and anatomy. Staining revealed large sensory cell bodies surrounded by satellite cells. Results are representative images of all animal groups (40X magnification).

**Fig 3:**
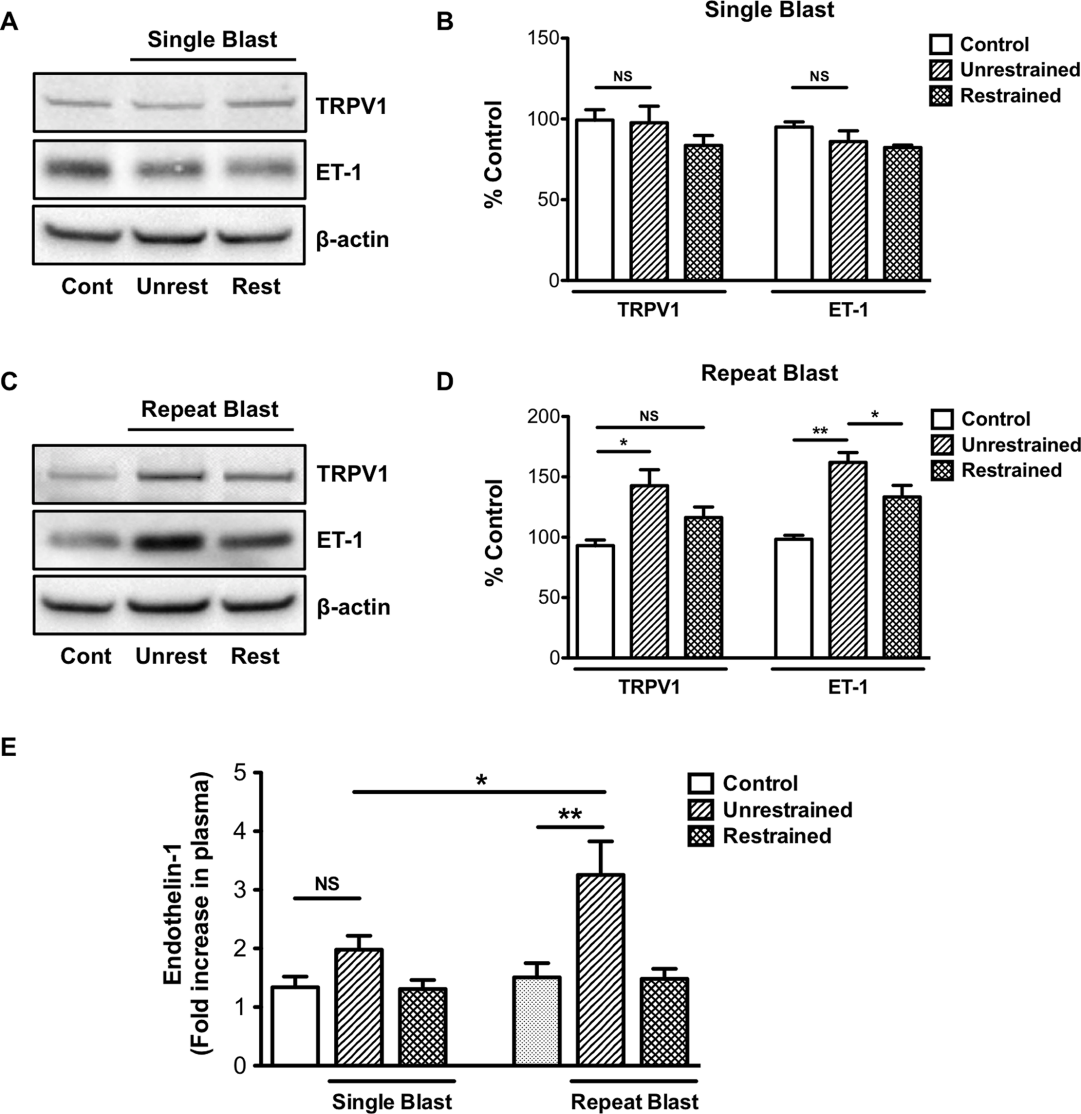
Repeat, tertiary low-level blast exposure increase TRPV1 and ET-1 expression in the trigeminal ganglion. Western blot analysis was performed on TGs harvested from restrained or unrestrained animals exposed to either a single or repeat, low-level primary or tertiary blast. (A, C) TGs were homogenized and whole cell lysates (50 μg) were assessed for TRPV1 and ET-1 protein expression. (B, D) Quantification of TRPV1 and ET-1 expression in unrestrained and restrained animals following exposure to a single or repeat blast, respectively. Results are representative of four independent experiments. (E) Quantification of ET-1 expression in blood plasma from all control and blast treatment groups. Data represented as fold increase in ET-1 expression as compared to control. NS, not significant, *p<0.05 and **p<0.01, one-way ANOVA with Tukey post-hoc test.

Recent findings in our laboratory showed that repeat low-level blast exposure resulted in increased TRPV1 and ET-1 expression and neutrophil infiltration in the rat cornea [10]. To expand on these data, we sought to investigate the effects of primary and tertiary low-level blast on ocular associated sensory tissues. For this study, animals that received a primary blast were restrained to the mesh holder, while animals that received a tertiary blast were unrestrained and allowed to recoil in response to blast (Fig 1B). Intraocular pressure (IOP) measurements taken before and after blast exposure did not reveal any differences in pressure among the animal groups (data not shown). As previously described [8–10], animals were exposed to a single or repeat low-level (73.8 ± 5.5 kPa) blast once or daily for five consecutive days. Following blast exposure(s), TG homogenates were prepared from all animals stained with H&E (Fig 2) to demonstrate ganglion structure/anatomy and also probed for TRPV1 and ET-1 protein expression (Fig 3). Increased TRPV1 or ET-1 expression was detected in unrestrained animals exposed to repeat, tertiary blast (Fig 3C and 3D); however no significant increases of these key proteins were detected in unrestrained or restrained animals that received a single low-level blast. Moreover, increased levels of ET-1 were also detected in the blood plasma from unrestrained animals exposed to repeat, tertiary blast as compared to single blast and control animals (Fig 3E).

### Repeat blast exposure results in increased TRPV1 and ET-A co-expression

**Fig 4:**
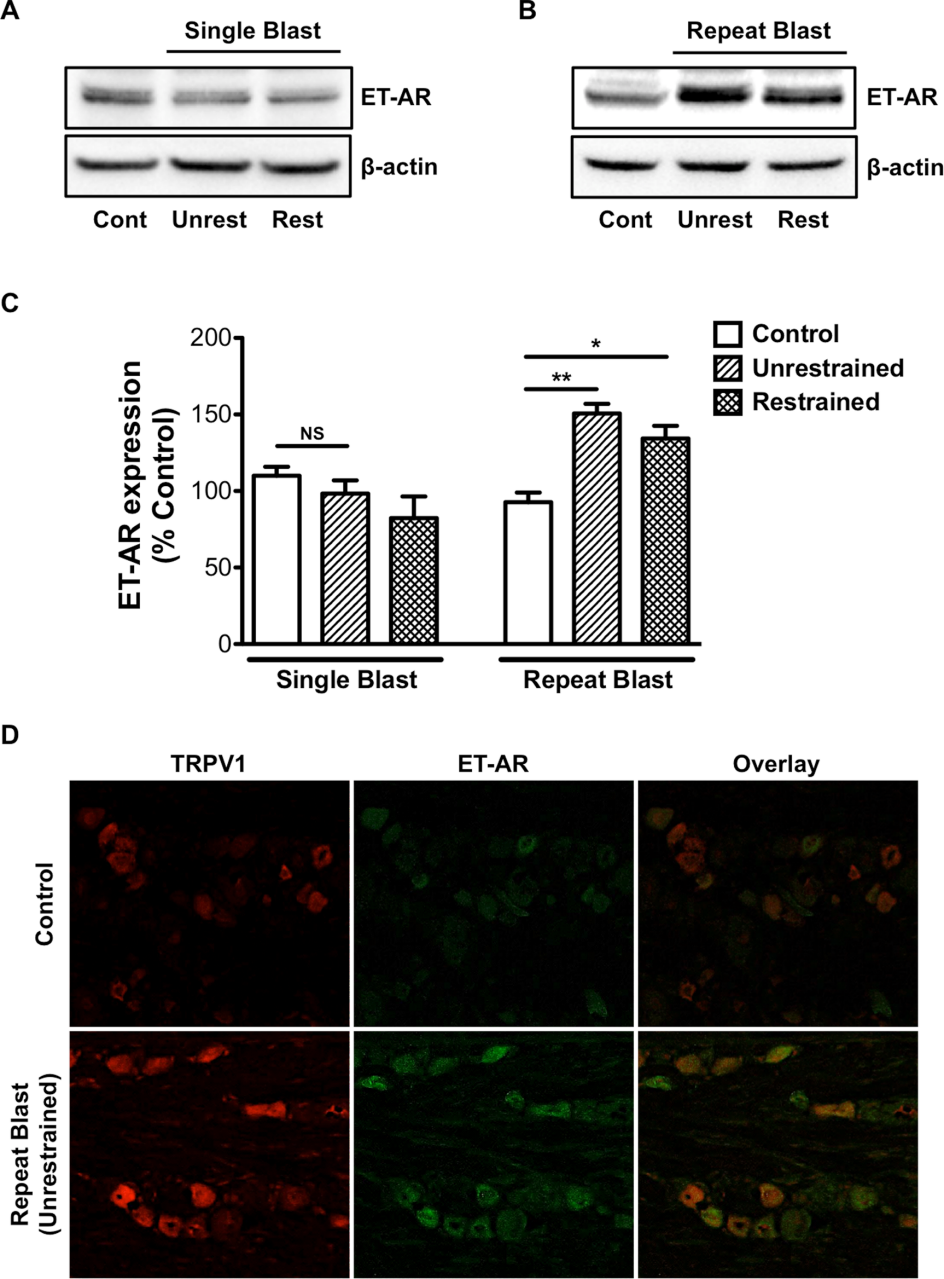
Repeat blast exposure results in increased TRPV1 and ET-A co- expression. Western blot and immunofluorescence analysis was performed on TGs harvested from animals that were exposed to either a single or repeat low-level primary or tertiary low-level blast. (A, B) TGs were homogenized and whole cell lysates (50 μg) were assessed for ET-A protein expression. (C) Quantification of ET-A expression in unrestrained and restrained animals following exposure to a single or repeat blast. (D) Immunofluorescence staining was completed on frozen sections of TGs subjected to repeat, tertiary blast. Tissues were subjected to staining with anti-TRPV1 (1:250) and anti-ET-A (1:250) antibodies and probed with Alexa Fluor 568 and 488 secondary antibodies, respectively. Representative images of an n = 4 for both control and repeat blast animals. NS, not significant, *p<0.05 and **p<0.01, one-way ANOVA with Tukey post-hoc test.

ET-1 is known to modulate TRPV1 activity via its ability to enhance receptor-mediated nociception and thermal hyperalgesic responses [12, 26–28]. The ET-1 receptor subtypes, ET-A and ET-B, are both expressed along the entire TG; however ET-A expression predominates within the three divisions of the TG [26] and is identified as the receptor subtype responsible for ET-1-mediated potentiation of TRPV1 [12, 13, 27]. Thus, we next examined the effect of primary and tertiary blast exposure on ET-A receptor expression in the TG. Increased ET-A receptor expression was observed in the TG from unrestrained animals subjected to repeat, tertiary blast as compared to single blast and control animals (Fig 4A and 4B). Increased expression of ET-A was not detected in unrestrained or restrained animals exposed to a single blast (Fig 4C). Immunofluorescence staining also revealed an increase in TRPV1 and ET-A co-expression in TGs harvested from unrestrained animals that received a repeat, tertiary blast (Fig 4D). No significant difference in TRPV1 and ET-A expression was observed in unrestrained or restrained animals exposed to a single, tertiary or primary blast, respectively (data not shown).

### Repeat, tertiary blast exposure results in enhanced nocifensive behavior

**Fig 5:**
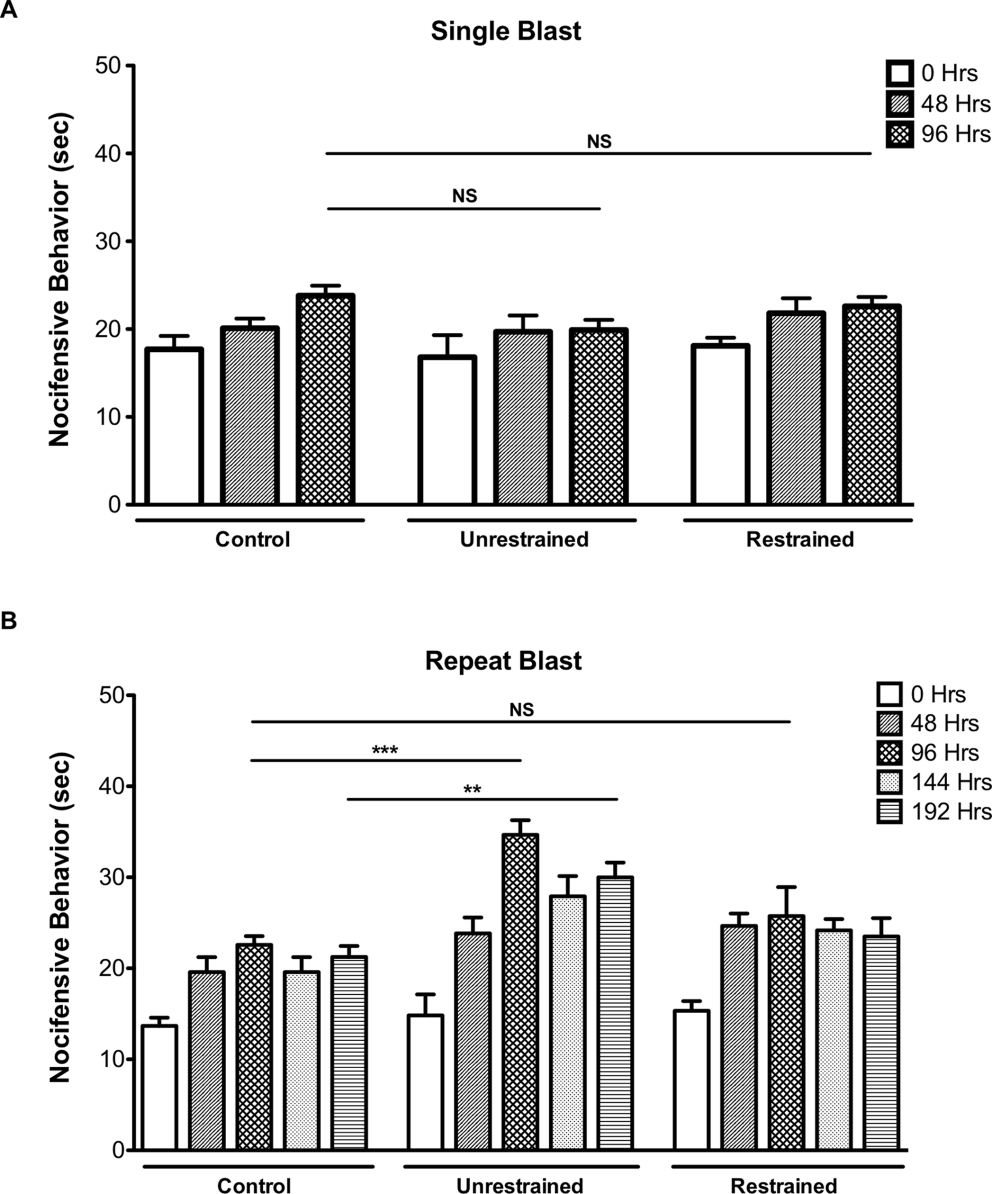
Repeat, tertiary blast exposure results in enhanced nocifensive behavior. A dilute concentration of CAP (0.02%) was applied directly to the eye of unrestrained and restrained animals exposed to primary or tertiary low-level single (A) or repeat (B) blast. Eye wipe (nocifensive) responses were recorded for two minutes following application of CAP. Responses were counted as the amount of the time animal spent flinching and wiping the affected eye with either the paw or hind leg. Behavioral assessments were conducted at the indicated time points. Data are representative of 5–6 animals per treatment group. NS, not significant, **p<0.01and ***p<0.001, Repeated Measures ANOVA with Tukey post-hoc test.

TRPV1, originally identified as the receptor for capsaicin (CAP), is a polymodal receptor with a dynamic threshold of activation that can be reduced under inflammatory conditions [29]. Since the TG provides dense sensory input to the cornea [30] and increased TRPV1 expression was found in the TG following repeat blast (Fig 3C and 3D) we next conducted nocifensive behavioral analysis. The eye-wipe test has been validated as a reliable method in trigeminal pain studies to assess TRPV1-mediated nocifensive behavior [24, 25, 31, 32]. Thus, to determine sensory activation following exposure to primary and tertiary blast, the eye wipe test was conducted in which a dilute concentration of CAP (0.02%) was applied directly to the eye (i.e. cornea) every 48 hrs. No difference in eye wipe (nocifensive) behavior was observed in unrestrained and restrained animals exposed to a single, primary or tertiary blast at the indicated time points (Fig 5A); however, unrestrained animals exposed to repeat, tertiary blast demonstrated a significant increase in nocifensive behavior as compared to control animals at 96 and 192 hrs post initial blast (Fig 5B). At the 96 hr time point, unrestrained animals exposed to repeat, tertiary blast spent an average of 35 sec versus 23 sec wiping and/or scratching the affected eye as compared to control animals. Moreover, this increase in nocifensive behavior persisted four days after the final blast (192 hr time point). No significant difference in nocifensive behavior was observed in restrained animals exposed to repeat, primary blast.

### Decreased TRPV1 desensitization in TG sensory neurons exposed to repeat, tertiary low-level blast

**Fig 6:**
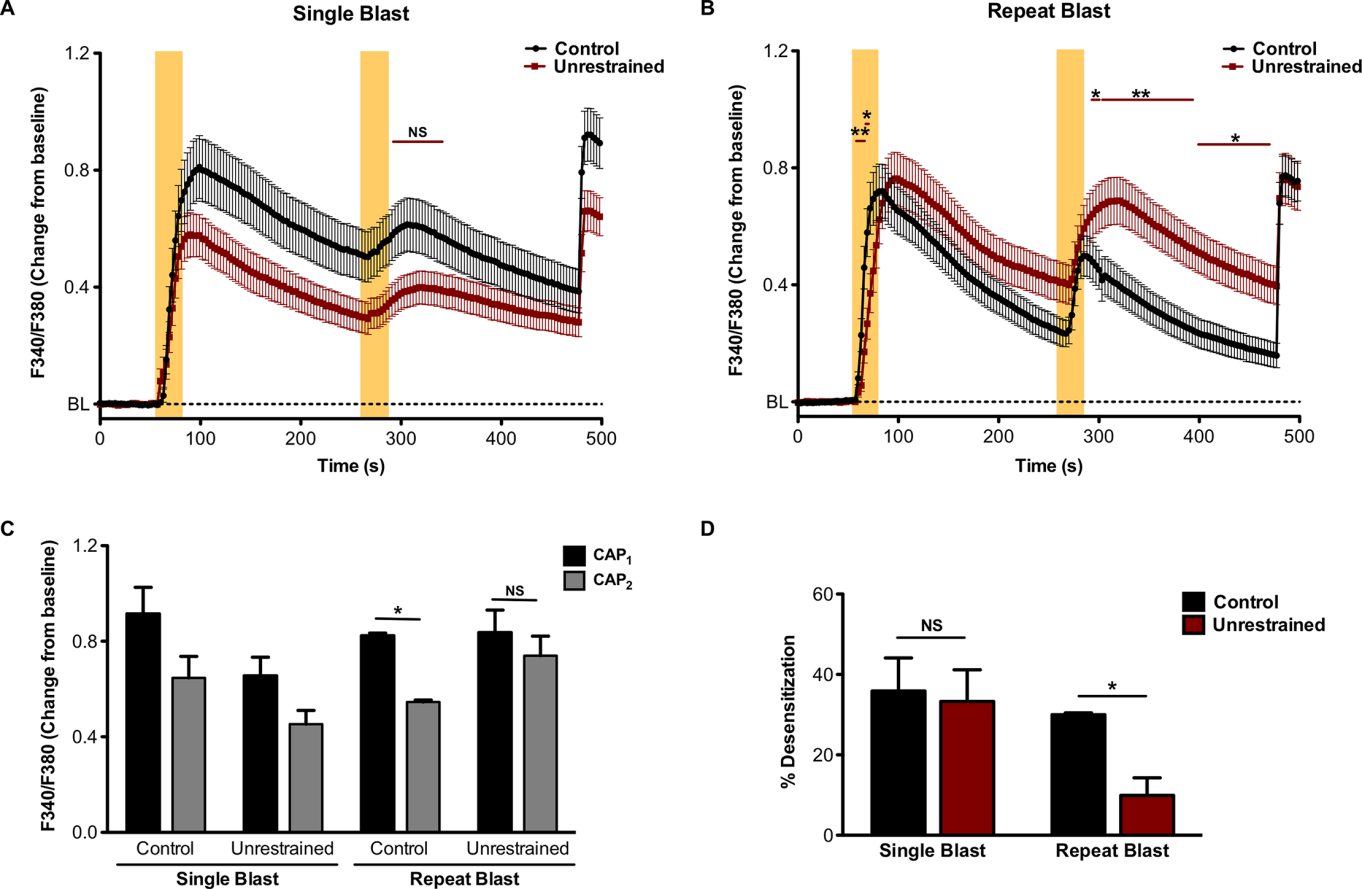
Decreased TRPV1 desensitization in TG sensory neurons exposed to tertiary low-level blast. TG were harvested from control animals and unrestrained animals exposed to a single or repeat, tertiary blast. Cumulative traces (A, B) and quantified responses (C) following initial and subsequent capsaicin (CAP) application, CAP_1_ and CAP_2_, respectively in KCl-sensitive TRPV1-expressing TG neurons. Shaded, yellow vertical bars denote application of CAP (50nM). (D) Quantified receptor desensitization of the indicated animal groups. NS, not significant, *p<0.05 and **p<0.01, two-way ANOVA with Bonferroni post-hoc test, n = 44–77 neurons/ group.

TRPV1 activation leads to a rise in cytosolic calcium that is subsequently followed by receptor desensitization, a state characterized by the inability of the receptor to respond to subsequent noxious stimuli [33]. However, following injury or trauma the release of inflammatory mediators function to increase receptor sensitivity and reduce calcium-dependent desensitization. To determine the functional consequences of repeat, tertiary blast on TRPV1 activity, we conducted calcium imaging on primary cultures of TG neurons harvested from animals exposed to a single or repeat, tertiary low-level blast. Calcium imaging analyses revealed that repeated application of CAP (50 nM, 30 sec) resulted in a significant decrease in receptor desensitization (Fig 6B and 6D) in TG neurons harvested from animals exposed to repeat, tertiary blast. No significant difference in TRPV1 desensitization was observed in TG neurons exposed to single, tertiary blast as compared to control animals (Fig 6A and 6C).

### Increased CGRP and SP expression in TG sensory neurons exposed to repeat, tertiary blast

**Fig 7:**
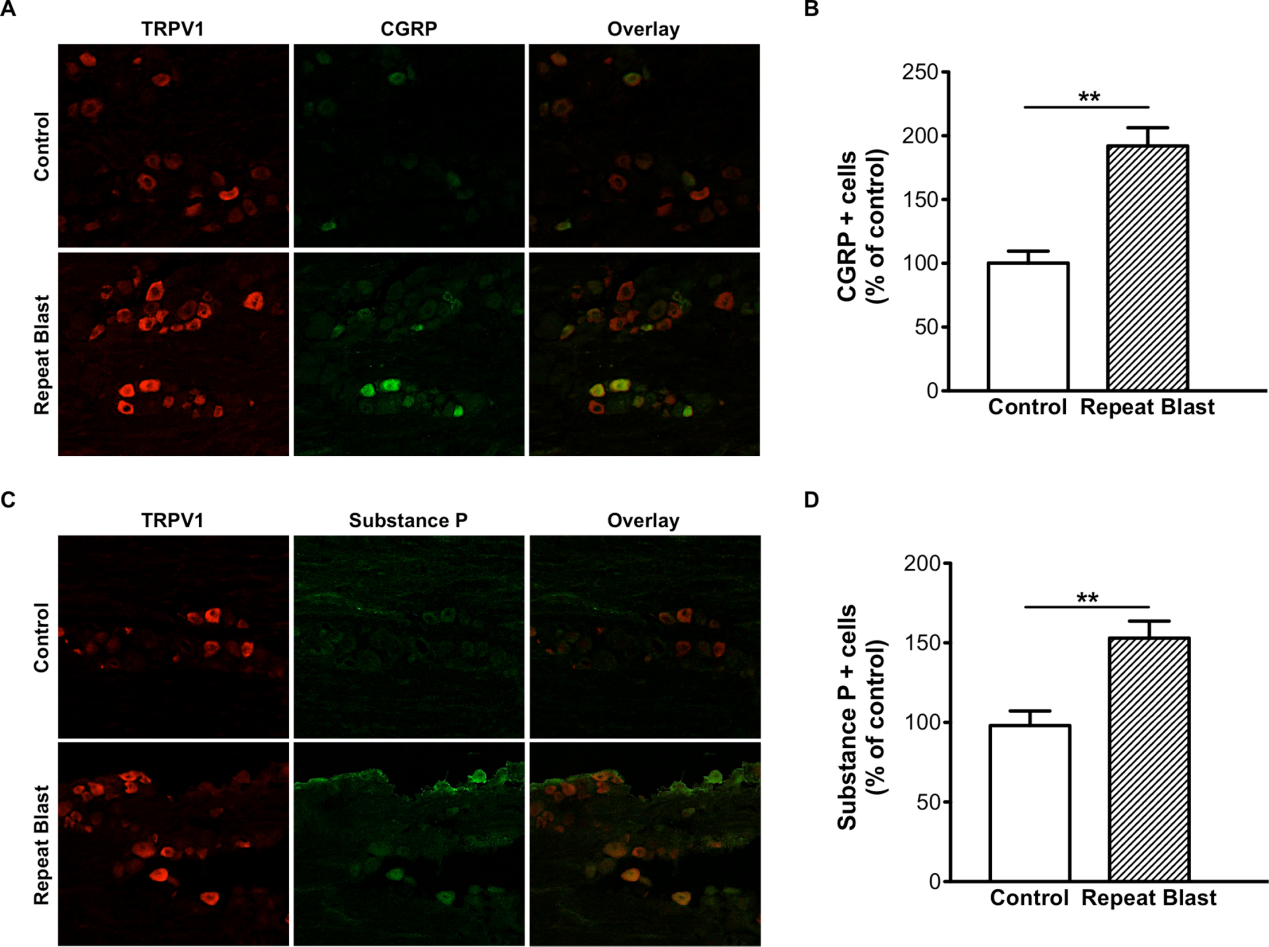
Increased CGRP and SP expression in TG sensory neurons exposed to repeat, tertiary blast. Immunofluorescence analysis was performed on frozen TG sections harvested from control and repeat, tertiary (unrestrained) blast exposed animals. Tissues were subjected to staining with anti-TRPV1 (1:250), anti-CGRP (A) (1:250) and anti-SP (C) (1:250) antibodies and probed with the appropriate Alexa Fluor 568 and 488 secondary antibodies. (B, D) Quantification of CGRP- and SP-positive cells as percent of control animals, respectively. Paired t-test, **p<0.01.

TRPV1 stimulation results in the activation of nociceptive inflammatory responses and the secretion of critical pain signaling neuropeptides, calcitonin gene-related protein (CGRP) and substance P (SP), from CAP-sensitive primary sensory neurons [34, 35]. CGRP and SP are simultaneously released from nociceptive C fibers and play important roles in the pathophysiology of various pain states [36]. As such, we also investigated the expression of CGRP and SP in the TG following low-level repeat blast exposure. TG sections prepared from control and repeat, tertiary blast exposed animals were stained with antibodies specific for CGRP and SP. Immunofluorescence staining revealed a signficant increase in CGRP and SP expression in repeat, tertiary blast tissues as compared to control (Fig 7B and 7D). No difference in expression was observed in restrained animals exposed to a single or repeat blast (data not shown). Furthermore, the increased expression of CGRP and SP was observed in TG neurons co-expressing TRPV1.

### Repeat low-level blast increases GFAP expression in the trigeminal ganglion

**Fig 8:**
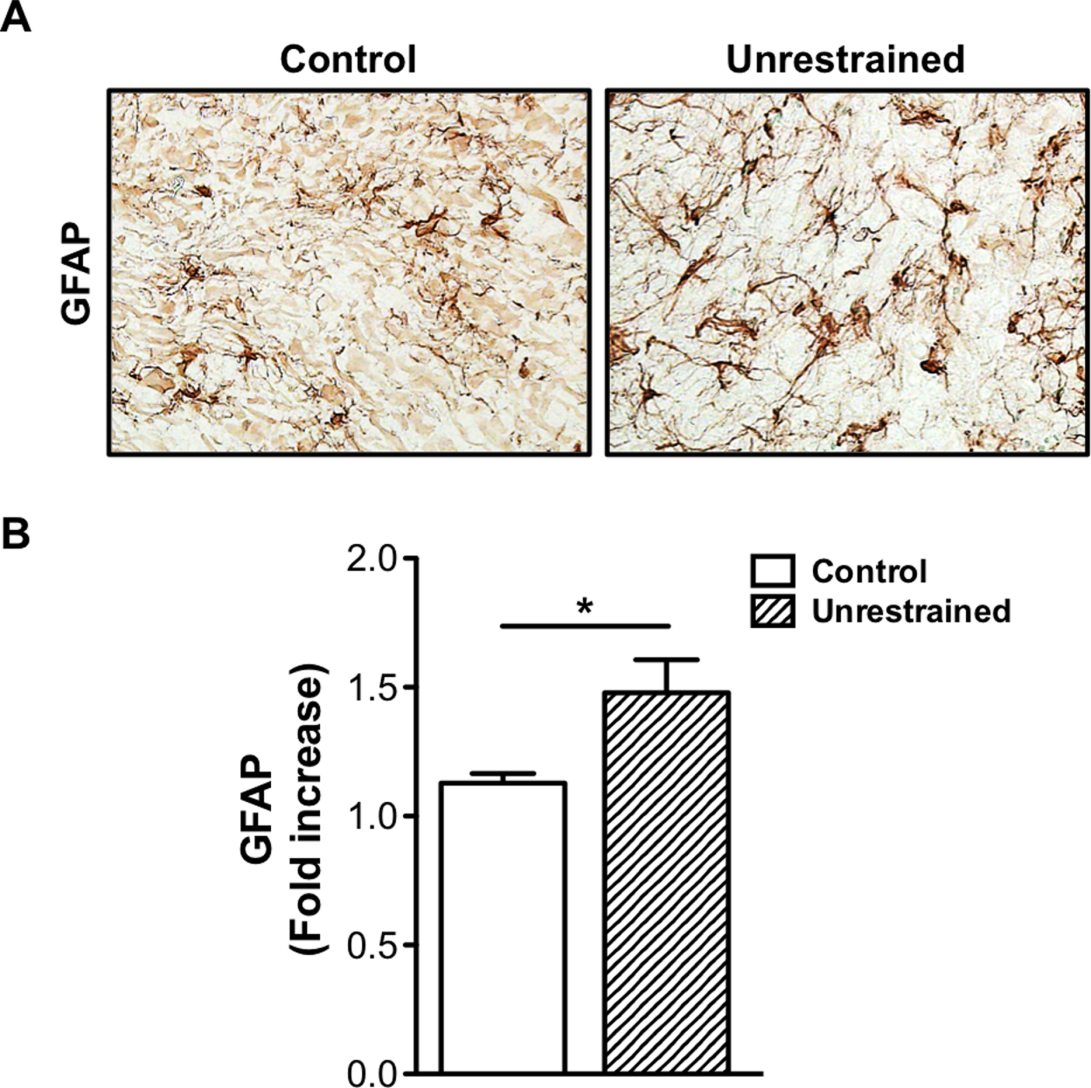
Repeat low-level blast increases GFAP expression in the trigeminal ganglion. (A) Immunohistochemical staining was performed on frozen TG sections harvested from control animals and unrestrained animals exposed to repeat, tertiary blast. Tissues were subjected to staining with anti-GFAP (1:1000) and then probed with the appropriate secondary biotinylated antibody. (B) Quantification of GFAP expression in TG homogenates from control and repeat, tertiary blast treatment groups. Data represented as fold increase in GFAP expression as compared to control. NS, not significant, *p<0.05, one-way ANOVA with Tukey post-hoc test.

Recent literature has shown that CGRP promotes cellular changes in TG sensory neurons and glia cells resulting in peripheral and central sensitization [37]. Satellite glial cells (SGCs) are prominent glial cells in the peripheral nervous system (PNS) and are found in the TG and dorsal root ganglion (DRG). Similar to astrocytes, SGCs express specific glial markers to include S100 and glial fibrillary associated protein (GFAP), an intermediate filament protein that is known to be up-regulated following trauma/injury and implicated in persistent pain [38, 39]. Thus, based on these evidence and our findings, which demonstrate increased CGRP expression in the TG, we next investigated whether repeat blast increased GFAP expression. Immunohistochemical staining conducted on TG sections prepared from animals exposed to repeat, tertiary blast revealed increased GFAP staining as compared to control (Fig 8A). This increase in GFAP protein expression was also confirmed and quantified from TG tissue homogenates (Fig 8B).

## Discussion

Repeated exposure to low-level blast is a characteristic of a few select occupations, to include specialized military and law enforcement units [40]. Specifically, with regard to military breacher training, personnel are typically exposed to 500–600 low-level blasts per year during their two to three training program [41]. Consequently, the polytrauma associated with blast injuries during military training and operations present serious challenges with respect to clinical assessments and rehabilitation efforts. Blast injuries to sensory systems, to include ocular and auditory, are among the most significant concerns of healthcare providers due to their high prevalence over the past few decades. In line with these concerns, recent literature has highlighted the negative effects of blast on visual and auditory function [9, 41–43] and provided additional insight into the pathophysiology of blast. The findings presented herein, provide further evidence of the effects of blast, namely on pain and inflammatory processes. Unrestrained animals subjected to repeat, tertiary blast showed significantly increased TRPV1 expression (Fig 3A and 3B), enhanced CAP-mediated nocifensive behavior (Fig 5B) and decreased receptor desensitization (Fig 6B and 6D) as compared to single, tertiary blast and controls. These data demonstrate the profound effects of blast on pain and inflammatory processes and highlight the trauma sustained are not due to the direct effects of the primary blast wave but rather from cumulative tertiary blast exposure associated with the movement of the head in response to the incident blast wave.

To date, multiple proteins and molecules are recognized for their ability to modulate TRPV1 activity and receptor desensitization [13, 44–50]. Of interest to our research, ET-1 enhances TRPV1-mediated nociception and thermal hyperalgesic responses [12, 26, 27]. ET-1 is a potent vasoconstrictor well characterized for its role in numerous vascular functions; however, research efforts are also focused on ET-1 as a pro-algesic/pain mediator and its contribution to chronic pain associated with a number of diseases [51–54]. Considerable evidence also implicates ET-1 in peripheral pain signaling, in that ET-1 injection leads to nociceptor excitation, induction of nocifensive behavior in animals and severe pain and tactile allodynia in humans [51–55]. TRPV1 knockout animal studies have also provided critical insight on ET-1-induced pain behavior and thermal hyperalgesia [11, 56]. To expand on this body of evidence, as well as our previous findings, which demonstrate increased ET-1 expression following repeat blast in ocular tissues [10], we herein show that repeat, tertiary blast increases ET-1 expression in the TG and blood (Fig 3C and 3E). Importantly, these increases were detected four days after the final blast, suggesting sustained ET-1 activation. Increased ET-A expression was detected in the TGs exposed to repeat, tertiary blast and was co-expressed with TRPV1 (Fig 5). These data indicate up-regulation of the ET-1 and ET-A signaling pathway following blast and provide some insight into the molecular mechanisms involved in enhanced pain signaling and/or processing.

ET-1-mediated PKC activation and subsequent TRPV1 phosphorylation is suggested as a mechanism that underlies ET-1 sensitization of nocifensive behavior and thermal hyperalgesia [13, 57]. Of the two ET-1 subtypes, ET-A is identified as the receptor responsible for ET-1-mediated potentiation of TRPV1 and is thought to contribute to the pain-producing and pain-potentiating effects of ET-1 [12, 13, 27]. To date, ET-A antagonists have been extensively studied as anti-cancer therapeutics for various malignancies with promising results [58–61]; however, recent animal studies have revealed that local administration of ET-A antagonists function to attenuate mechanical allodynia and thermal hyperalgesic responses [62–64]. Unfortunately, considerable research on TRPV1 antagonists as therapeutic analgesics has been unsuccessful due to severe side effects, most notably hyperthermia [65, 66]. Thus, ongoing pain research is currently focused on the contribution of endogenous molecules and proteins involved in the regulation of TRPV1. Pharmacological compounds that indirectly attenuate TRPV1-mediated pain and associated signaling pathways, without adverse side effects, are of great interest for clinical translation. As such, future studies are necessary to determine the safety and efficacy of ET-A antagonists in the treatment of painful disease states and conditions that are known to be associated with increased circulating ET-1 levels.

Extensive research has established the fundamental role of TRPV1 and associated neuropeptides, to include CGRP and SP, in nociceptive signaling and the progression of chronic, persistent pain. Of relevance, recent data have implicated increased CGRP levels in peripheral and central sensitization [37], whereas increased expression functions to enhance nocifensive responses via protein kinase A (PKA) and GFAP associated mechanisms, suggesting neuron and glial communication in these processes [67]. GFAP, a biomarker of astrocyte activation, belongs to a heterogeneous group of intermediate filaments that is expressed in mature astrocytes and is known to be up-regulated in response to peripheral nerve damage [39]. Following trauma or injury, increased GFAP expression in SGCs and release of various inflammatory mediators has been shown to potentiate inflammation and hyperalgesia [38, 68]. Previous studies in our laboratory have shown that repeat low-level blast results in increased expression of GFAP and CD68, markers for gliosis and inflammation [8]. Activation of glial cells and neuro-glial interactions following trauma/injury are emerging as key mechanisms underlying the development and maintenance of chronic pain. These interactions have also been shown to exhibit enhanced coupling in persistent inflammatory and neuropathic pain [39, 69] and thought to underlie activity dependent plasticity as well [38]. Collectively, these findings support a role of glia and cytokines in persistent pain in the TG. To comprehensively delineate blast-associated pain and inflammatory processes, future studies are required to expand upon this knowledge base and appropriately evaluate neuronal and glial signaling pathways in sensory tissues.

In summary, repeat tertiary blast exposure increased TRPV1, ET-1 and ET-A receptor expression in the trigeminal ganglion. Cumulative, repeat low-level blast in our rodent model also resulted in increased nocifensive behavior and decreased TRPV1 desensitization, which was further supported by increased expression of the neuropeptides, CGRP and SP. Increased GFAP expression in TGs from unrestrained animals exposed to repeat, tertiary blast also demonstrated blast effects on neuron and glial cell activation. Taken together these data provide critical insight into blast injury/trauma with respect to pain and inflammatory processes and may serve to identify therapeutic targets for blast-associated injuries. Pharmacological inhibitors or receptor antagonists that indirectly modulate TRPV1 activity and desensitization deserve consideration as potential treatments for chronic pain and/or inflammation associated with this complex trauma.

## Supporting information

S1 Supporting Document Data files for Figs 3–8. (XLSX)
